# On the Possibility of retrograde calculation of blood alcohol concentrations below 0.15‰

**DOI:** 10.1007/s00414-026-03838-6

**Published:** 2026-05-28

**Authors:** Ava T. R. Mihan, Stefan Toennes, Alexander Paulke, Marcel A. Verhoff

**Affiliations:** Institute of Legal Medicine, University Hospital, Goethe University Frankfurt, Kennedyallee 104, Frankfurt am Main, D-60596 Germany

**Keywords:** Blood alcohol, Minimum blood alcohol concentration, Retrograde extrapolation, Ethanol elimination, Forensic toxicology

## Abstract

It is widely recognised in forensic medicine that retrograde extrapolation of blood alcohol concentrations (BAC) is generally avoided once the ethanol concentration falls below 0.15‰. In Germany, retrograde calculation is commonly applied to estimate a minimum BAC in favour of the subject and a low elimination rate of 0.1‰/h is legally accepted; however, expert practice typically refrains from back-calculation when the measured BAC is below 0.15‰ *(“Rückrechnungsverbot”)*. This pragmatic threshold is rooted in historical concerns about uncertainty near the end of the BAC curve and the increasing influence of analytical and kinetic variability at very low concentrations. A re-analysis was conducted using datasets from four controlled drinking trials (n = 87), in which measurements were continued until complete ethanol elimination. The analysis demonstrated that a linear relationship of ethanol elimination persists below the 0.15‰ threshold and that elimination kinetics remain largely unaffected by the type of alcoholic beverage consumed or by the concentration intervals examined (0.3-0‰; 0.6 − 0.3‰; 0.9 − 0.6‰; cmax-0.6‰; cmax-0.9‰). Algorithms were developed to perform regression analyses across these distinct segments of the blood alcohol concentration curve and to derive parameters for an overall linear relationship from cmax to 0.0‰. The arithmetic mean of the average elimination rates – both across different beverage types and across the respective BAC intervals – was approximately 0.13‰/h. Within the 0.3-0‰ segment, all mean elimination rates exceeded 0.11‰/h. The integrative algorithmic analyses further indicated that the slope representing ethanol elimination below the 0.15‰ threshold averaged approximately 0.083‰/h, remaining relatively close to the conventionally applied minimum rate of 0.1‰/h. Based on these findings, an optimised rule for retrograde extrapolation was developed. The modified extreme model for BAC values ≤ 0.15‰ allows a simple and practicable estimation of minimum BAC by applying the low elimination rate of 0.1‰/h after subtracting a corrective time adjustment dependent on the initial measured BAC. To ensure forensic reliability, initiation of retrograde extrapolation from a conservative lower threshold of 0.06‰ is recommended. Hence, as an alternative strategy for calculating the minimum BAC at the time of the incident, it is proposed that, for measured BAC values between 0.06 and 0.14‰, a one-time deduction of 43 min be subtracted from the conventional retrograde calculation time interval, after which back-calculation may proceed using an elimination rate of 0.1‰/h. Retrograde extrapolation should then be omitted entirely for measured BAC values below 0.06‰.

**Supplementary Information:**

The online version contains supplementary material available at 10.1007/s00414-026-03838-6.

## Introduction

In Germany and many other countries, retrograde extrapolation of blood alcohol concentration (BAC) is a routine task in legal medicine, forensic toxicology, and traffic medicine. But, its permissibility and practical handling at very low measured BAC values differs between jurisdictions and expert traditions. In some settings, extrapolation is discouraged or avoided below a defined threshold, because close to zero it becomes increasingly difficult to exclude late-phase elimination behaviour and because relative analytical uncertainty becomes more influential. As a result, borderline cases may be handled inconsistently, despite the fact that such low BAC values can still be relevant in legal contexts (e.g., assessment of recent drinking, plausibility checks of statements, or interpretation of marginal findings).

Retrospective estimation of BAC has traditionally been performed using a linear (Widmark-type) elimination assumption; however, elimination kinetics are not strictly constant across the concentration-time curve and are influenced by the concentration range considered and by absorption conditions. In particular, when calculations are started from very low BAC values, both kinetic-model uncertainty and analytical imprecision become increasingly influential, which may affect the robustness of retrospective estimates.

In forensic expert practice in Germany, retrograde calculation of the minimum BAC is generally not considered reliable when the initially measured value is below 0.15‰. The present study addresses this practical gap by re-analysing controlled drinking data with measurements extended to complete ethanol elimination. We quantify elimination behaviour in the low-BAC range and derive a conservative, easy-to-apply correction approach for retrograde estimation below 0.15‰, including a suggested lower limit for application.

A review of the scientific basis commonly cited for omitting retrograde extrapolation at very low BAC values indicates that several practical rules originate from experimental work in which elimination rates were estimated over specific post-peak concentration ranges and observation windows. Because the apparent slope of the concentration-time curve depends on the concentration interval considered and on absorption conditions, elimination estimates may vary and should be applied with caution when calculations are initiated from very low BAC values (Lewis 1986; Mihan et al. 2025) [1, 2].

At present, gas chromatography is the method most widely used. According to the “Guidelines for determining blood alcohol concentration (BAC) in blood for forensic purposes,” jointly issued by the German Society of Legal Medicine (DGRM), the German Society of Toxicological and Forensic Chemistry (GTFCh), and the German Society for Traffic Medicine (DGVM) [3], determination of BAC in criminal proceedings requires measurement of the ethanol concentration in whole blood (or measurement in serum/plasma with subsequent conversion of the result to an equivalent whole-blood concentration) using two different analytical methods, each performed in duplicate. The legally relevant BAC corresponds to the mean value of the four individual measurements. The range of values must not exceed 10% of the mean value (for mean values < 1‰, a maximum deviation of 0.1‰ is allowed). Calculating the mean of four individual determinations increases statistical reliability and compensates for random measurement errors. To ensure and verify reproducibility and accuracy, regular calibrations and controls using quality-assured samples (internal quality control) as well as proficiency testing schemes (external quality control) are routinely performed.

Controlled drinking studies with serial sampling continued to complete ethanol elimination permit a direct evaluation of elimination behaviour in the low-BAC range, including the interval below 0.15‰. For this purpose, the raw data of four studies of controlled drinking experiments, carried out at the Frankfurt Institute of Legal Medicine, were pooled and analysed. Blood sampling was continued until the end of the elimination curve (at least until a negative breath alcohol test was obtained). While the general principles of post-peak BAC decline are well described, practical back-calculation at very low measured BAC values remains debated, because uncertainty increases as concentrations approach zero and absorption-related effects can influence the curve near the peak. The focus of the present study was to investigate alcohol elimination during the final phase of the curve, and to assess the elimination process in this range. The central question was whether, in cases of measured BACs below 0.15‰, retrograde calculation of the minimum BAC for the time of the incident might be possible – and if so, under which prerequisites or conditions.

## Methods

The underlying experimental datasets were derived from four previously published controlled drinking studies conducted at the Institute of Legal Medicine, Goethe University Frankfurt am Main, whose data were pooled as the primary empirical basis for the original regression-based modelling, Monte Carlo simulation, and derivation of the forensic correction algorithm presented herein. The studies involved healthy adult volunteers who provided written informed consent, and all studies were approved by the responsible ethics committee. Participants consumed alcoholic beverages comprising wheat beer (with and without additional administration of the purported sobering agent “Eezup!”), apple wine (commercially available and self-produced), grappa, and vodka, as described in detail elsewhere [4–7]. Where specified in the original protocols, sobriety was confirmed prior to alcohol administration, and participants received a standardised meal to minimise variability in absorption conditions. Alcohol doses were individually calculated based on sex and body weight using Widmark-based approaches, with adjustment for presystemic elimination/resorption deficit, targeting peak blood alcohol concentrations of approximately 1.0‰ under controlled conditions, and blood sampling was continued until complete ethanol elimination. The original study designs did not include stratification based on individual alcohol tolerance; this reflects a heterogeneous volunteer sample rather than a preselected tolerance group and is compatible with the forensic aim of developing a robust rule applicable to inter-individual variability in practice.

Overall, data from 87 participants were included. As the administration of “Eezup!” showed no statistically significant effect on BAC elimination [4], the corresponding datasets were pooled without modification. For quantitative evaluation of ethanol elimination in the low-concentration range, all individual post-absorptive BAC-time curves were systematically screened for data points ≤ 0.15‰. For each subject, the elimination phase was defined from the last occurrence of the individual peak BAC (c_max_) to the end of the measurement series. Within this phase, all measured values ≤ 0.15‰ were included. In total, 459 individual post-peak data points ≤ 0.15‰ were available for analysis.

BAC-time data were analysed using linear regression models to characterise ethanol elimination. For each individual curve, the post-absorptive phase was identified, with the upper limit defined as the maximum measured BAC (c_max_) and the lower limit as the first zero or near-zero measurement. Linear regression was applied to the full elimination phase (c_max_-0‰) as well as to predefined sub-intervals ([0.9 − 0.6‰], [0.6 − 0.3‰], [0.3-0‰], [c_max_-0.6‰], and [c_max_-0.9‰]). The resulting slope coefficients represent individual ethanol elimination rates expressed in ‰/h.

All analyses were primarily performed using Python (*pandas*,* NumPy*,* scikit-learn*). To ensure robustness and reproducibility, regression analyses were independently cross-checked using Microsoft Excel (LINEST function).

In a second analytical step, simulation-based retrograde calculations were performed to develop practical back-calculation models for low measured BAC values (< 0.15‰). Three approaches were evaluated: a *conservative model*, a *realistic model*, and an *extreme model*. In all models, back-calculation was based on a standard elimination rate of 0.1‰/h; in the realistic and extreme models, additional time deductions were applied to account for reduced elimination rates in the low-BAC range.

As a reference, the uncorrected linear *conservative model* for determination of the minimum BAC was used, which back-calculates using a low constant elimination rate of 0.1‰/h over the time interval *t* between blood sampling and the incident time:

$$BAC_{retrograde}=BAC_{measured}+0.1\bullet t$$

The realistic model was derived from mean elimination rates observed below 0.15‰, incorporating confidence interval-based adjustments to account for inter-individual variability. The extreme model was based on the lowest empirically observed elimination rate to ensure maximal protection against overestimation.

Model robustness was assessed using Monte Carlo simulations (1,000 iterations per BAC range), generating normally distributed elimination rates based on the observed mean and standard deviation. Resulting distributions of back-calculation time deductions were evaluated to quantify uncertainty and validate model stability.

## Results and discussion

### Ethanol elimination with particular consideration of low BAC values

The hourly rates of blood alcohol concentration (BAC) decrease across the different studies and across the various sections of the concentration–time curves are summarised in Table 1.

**Table 1:** Hourly rates of blood alcohol concentration (BAC) decrease in the different studies and across various sections of the concentration–time curves

Interval	Wheat beer [‰/h]	Wheat beer + Eezup! [‰/h]	Apple wine [‰/h]	Grappa [‰/h]	Vodka [‰/h]
c_max_ – 0‰	0.143	0.150	0.162	0.168	0.159
0.3–0.0‰	0.111	0.126	0.142	0.143	0.125
0.6–0.3‰	0.162	0.175	0.174	0.182	0.184
0.9–0.6‰	0.110	0.109	0.163	0.143	0.139
c_max_ – 0.6‰	0.105	0.104	0.155	0.135	0.133
c_max_ – 0.9‰	0.078	0.102	0.121	0.154	0.069

The overall hourly decline in blood alcohol concentrations across the full range [c_max_–0‰] was 0.156‰/h, which is consistent with the average elimination rate reported in the literature [8].

Analysis of the 75 data sets in the range below the 0.15‰ threshold (linear regressions of the subsection [0.15–0‰]) yielded a mean decline in BAC of 0.083‰/h (Supplementary Table S1). This value lies below the rate of 0.1‰/h commonly regarded as the minimum elimination rate and is clearly lower than the elimination rate observed across the entire BAC-time curve [c_max_–0‰] (Supplementary Table S2)*.*

**Fig. 1:**
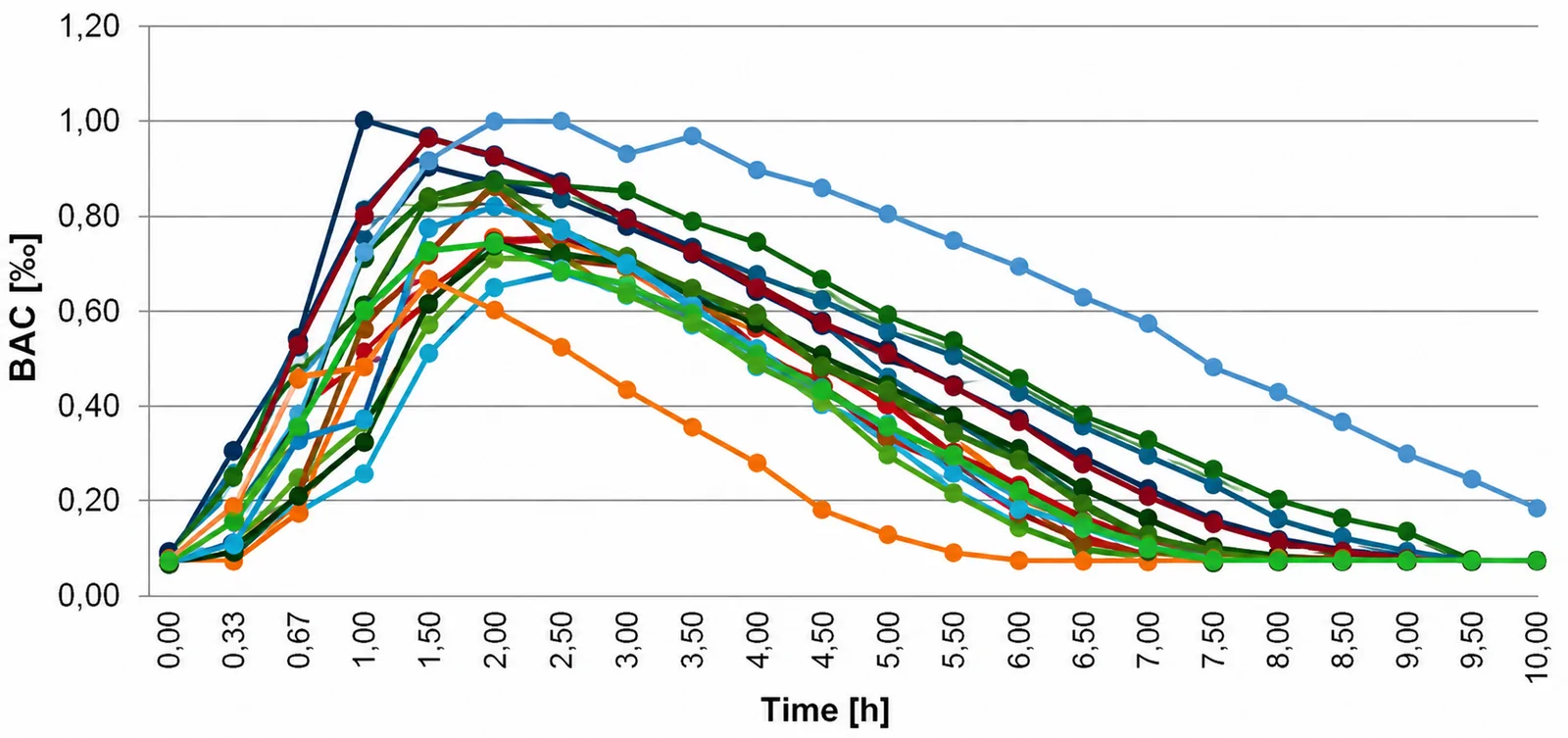
Blood alcohol concentration-time curve (wheat beer)

**Fig. 2:**
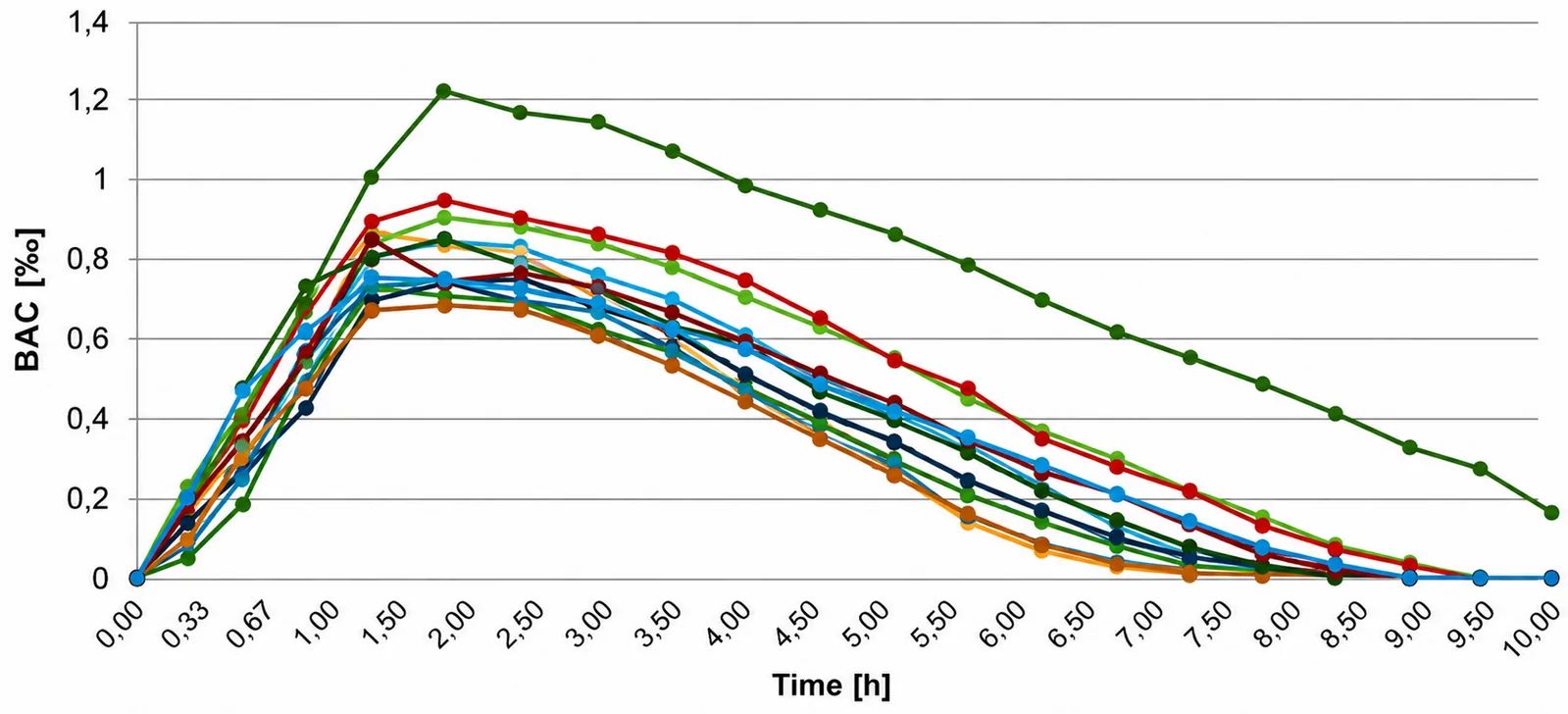
Blood alcohol concentration-time curve (wheat beer + Eezup!)

**Fig. 3:**
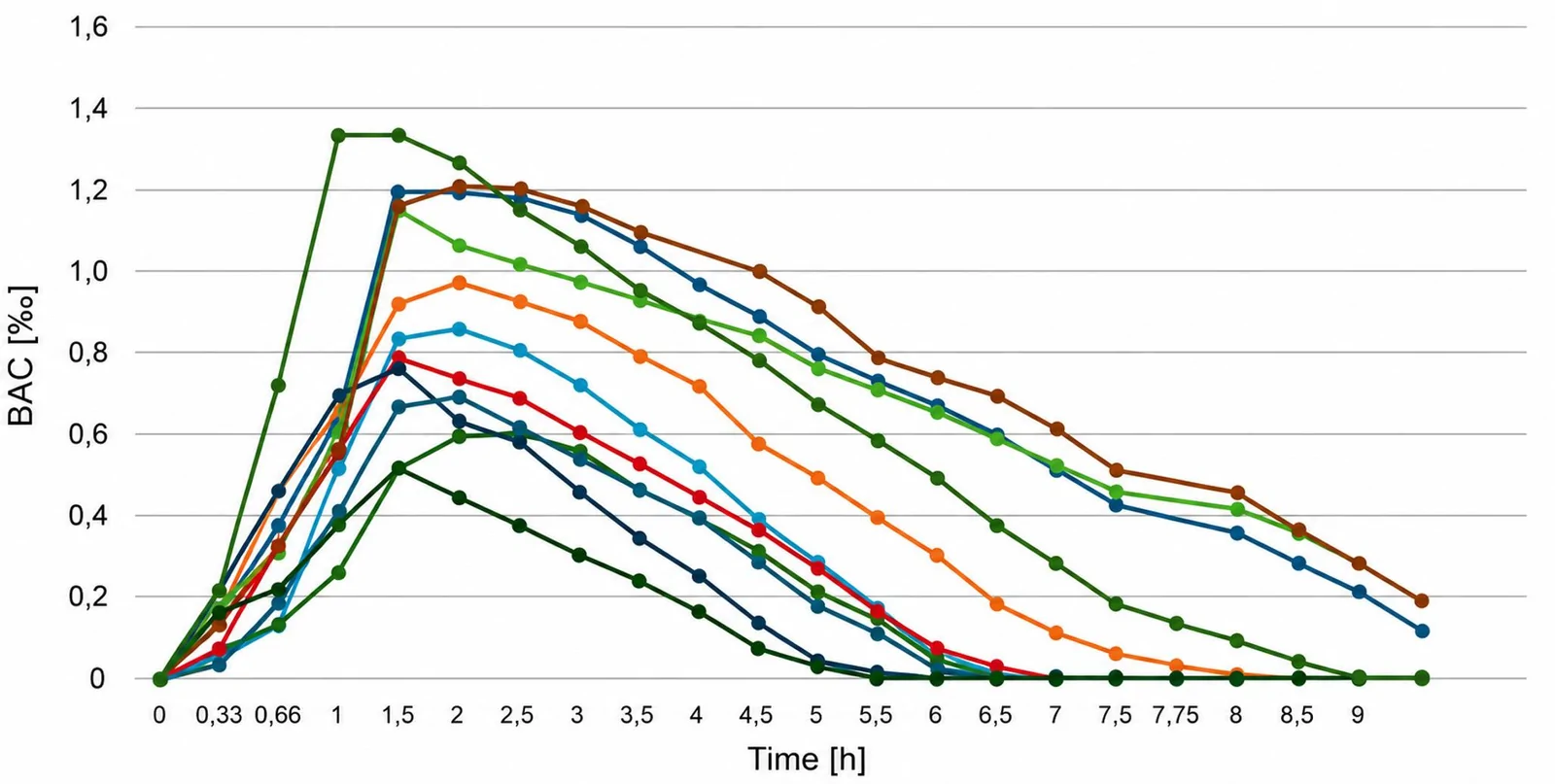
Blood alcohol concentration-time curve (apple wine, homemade)

**Fig. 4:**
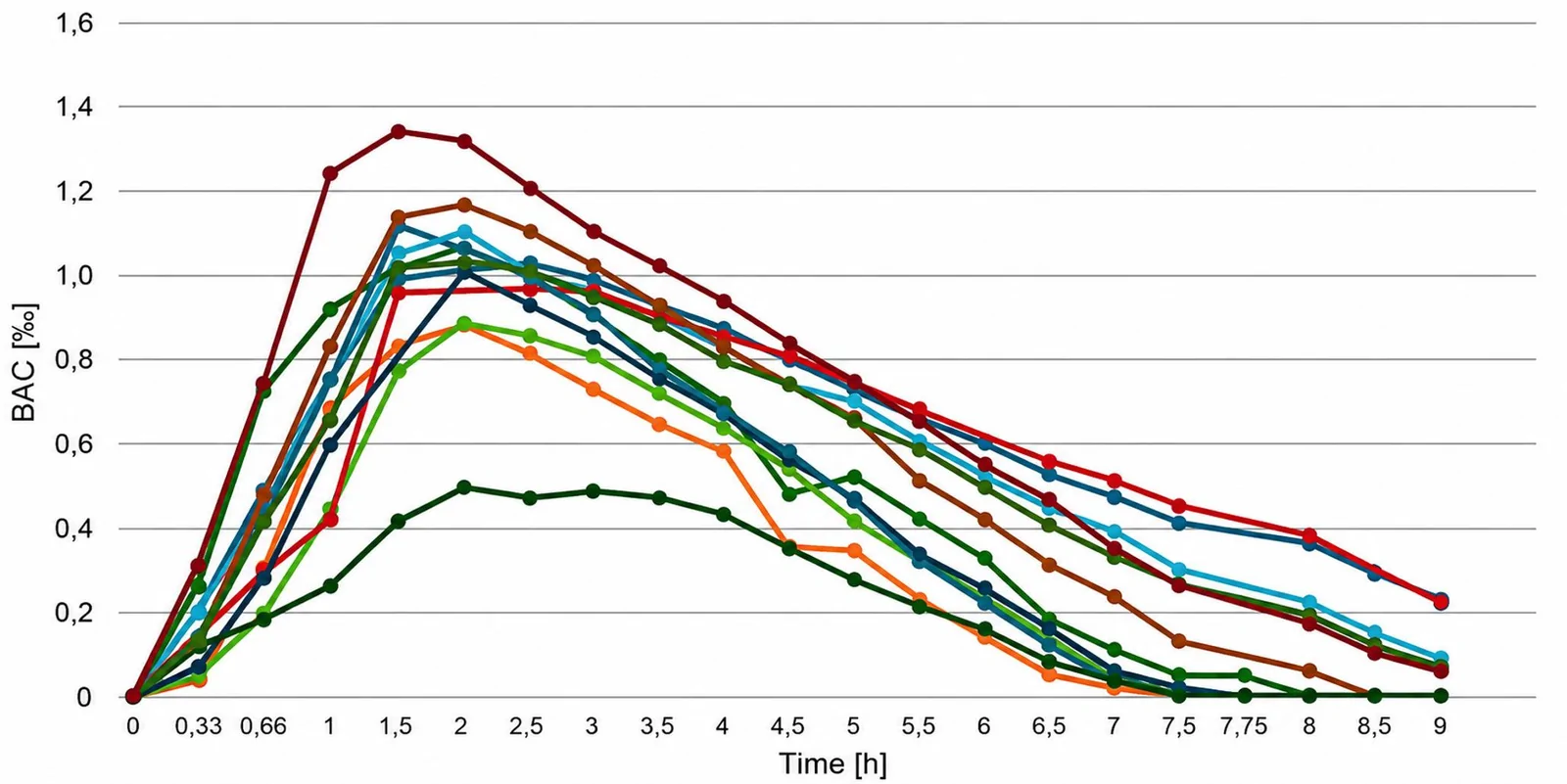
Blood alcohol concentration-time curve (apple wine, commercial)

**Fig. 5:**
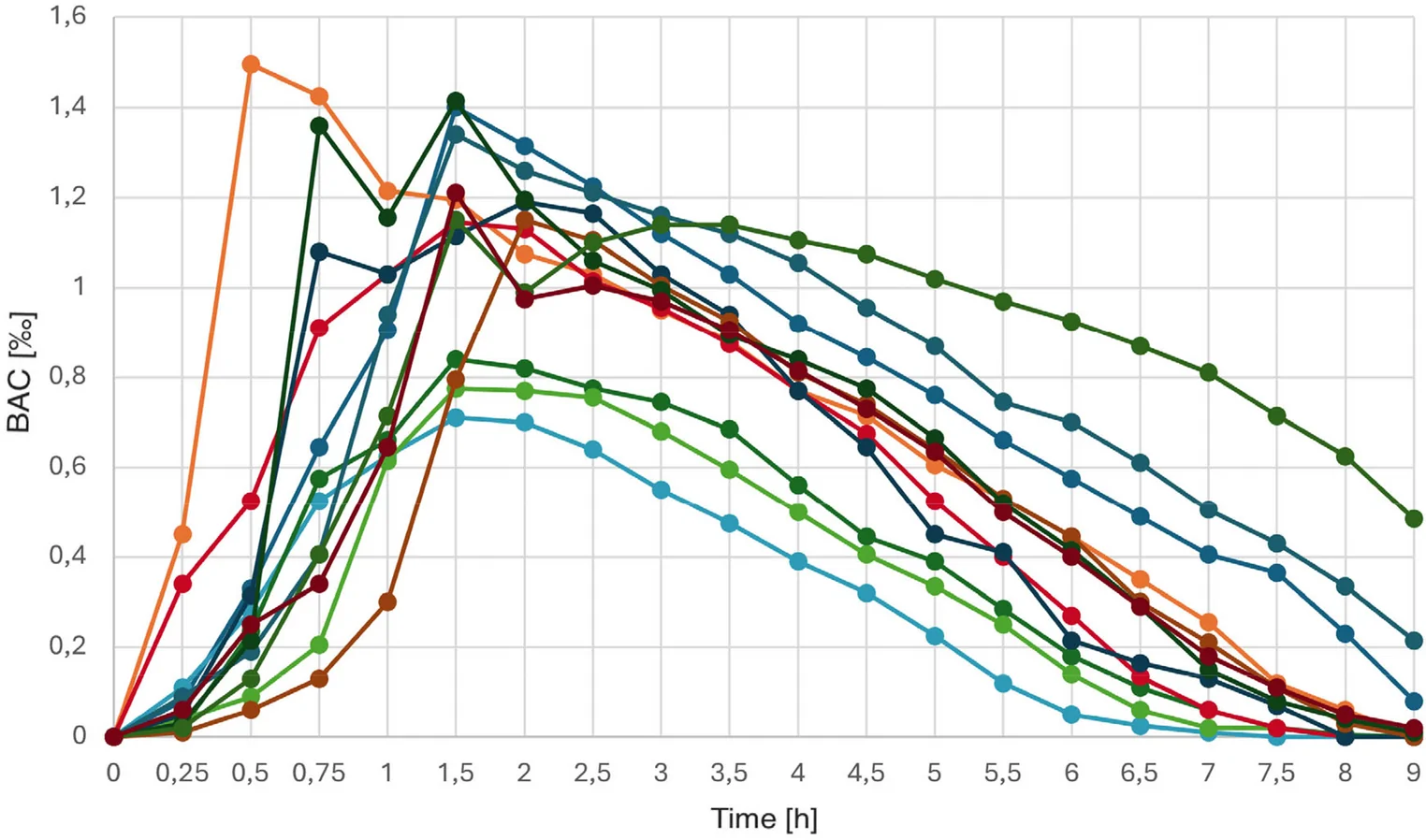
Blood alcohol concentration-time curve (grappa)

**Fig. 6:**
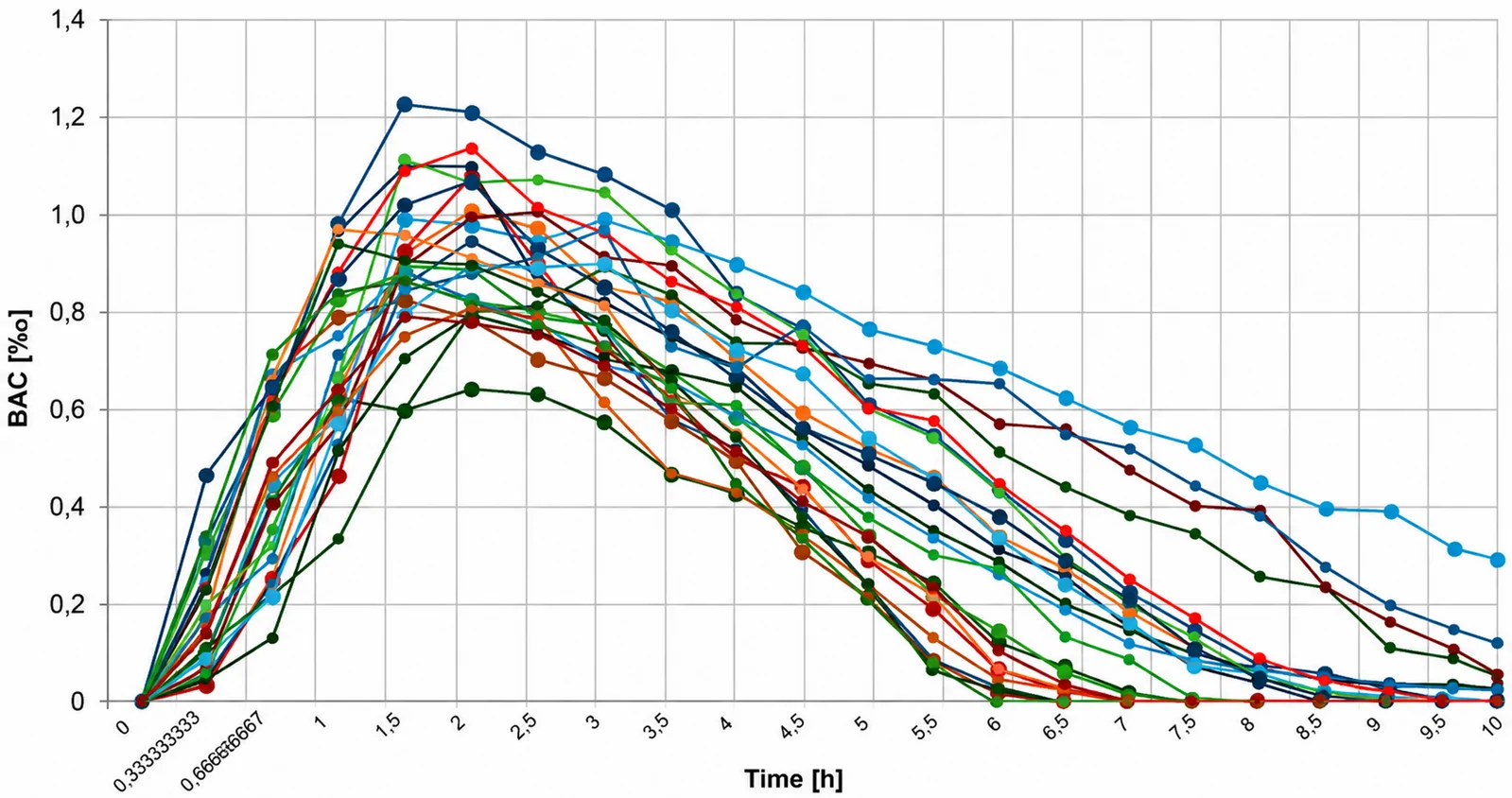
Blood alcohol concentration-time curve (vodka)

Closer inspection of the calculated elimination rates (Supplementary Table S2) initially reveals that elimination velocities were broadly comparable across all regression ranges of the BAC-time curves, irrespective of the type of alcoholic beverage. Wheat beer (Fig. 1) showed a decline of 0.11‰/h; the same beverage combined with “Eezup!” (Fig. 2) exhibited a nearly unchanged rate of 0.12‰/h. The arithmetic means for both self-produced (Fig. 3*)* and commercially available apple wine (Fig. 4), as well as for grappa (Fig. 5), amounted to 0.15‰/h, while vodka (Fig. 6) showed a rate of 0.13‰/h. Apple wine and grappa yielded the highest average elimination rates, whereas wheat beer showed the lowest.

In the interval of particular interest, between 0.3 and 0‰, the overall mean of all elimination rates was approximately 0.13‰/h. Elimination rates appeared consistent within this interval and were of the same order of magnitude as those observed in higher intervals (e.g. c_max_–0.6‰). This was confirmed by the ANOVA including all five groups (F = 1.64, *p* = 0.204 > 0.05), indicating no statistically significant differences between the groups, which may therefore be considered statistically homogeneous. As expected, this conclusion is further supported by the low overall dispersion of all values (standard deviation = 0.0309, corresponding to ~ 24% of the mean; range = 0.1149). These findings support continued mathematical application of a linear relationship in this segment of the BAC curve.

In the higher ranges of the BAC-time curves, elimination rates originating from c_max_ appeared lower than the average across the entire curve, which can be explained by the transition from the absorption phase to the elimination phase. Thus, mean elimination rates in the ranges 0.9–0.6‰ and c_max_–0.6‰ were approximately 0.13‰/h, while only the subsection 0.6–0.3‰ showed a slightly higher mean of approximately 0.18‰/h. This demonstrates a degree of consistency that is reliable for established back-calculation rules. In principle, all observed average elimination rates exceeded 0.1‰/h. Taken together, the modal value and median of approximately 0.13‰/h may therefore be regarded as the most suitable descriptive parameter for ethanol elimination velocity overall, particularly given that this value consistently represents both the arithmetic mean across all subdivided segments of the BAC curve and the cross-sectional mean across all beverage types.

Overall, with respect to the research question, it can be concluded that ethanol elimination rates in the range 0.3–0‰ (mean ≈ -0.129‰/h), which corresponds to measured initial BACs below 0.15‰, did not fall significantly below those of other intervals of the elimination curve (mean x̄ ≈ -0.1375‰/h, SD = 0.0248‰/h, minimum = -0.1755‰/h, maximum = -0.1048‰/h). To verify this, a Grubbs test (G test) for identification of outliers in a small normally distributed sample was applied. In the present case, no outlier was detected, as G < G_crit_ (Grubbs statistic G = 0.327; critical value G_crit_ ≈ 1.89 for α = 0.05, *n* = 6).

No statistically significant differences were detected between elimination behaviours of the different types of alcohol. The Grubbs significance test (α = 0.05, two-sided) yielded a mean x̄ ≈ -0.1337‰/h, SD = 0.0170‰/h, minimum = -0.1513‰/h, maximum = -0.1132‰/h, Grubbs statistic G = 1.2046, and critical value G_crit_ = 1.7150; since G < G_crit_, no significant outlier was present.

Across the various BAC intervals and beverage types, ethanol metabolism rates remained largely consistent, with no substantial differences between elimination at higher and lower blood ethanol levels that would be statistically significant or relevant for back-calculation in favour of the defendant (mean x̄ ≈ -0.1375‰/h, SD = 0.0248‰/h, minimum = -0.1755‰/h, maximum = -0.1048‰/h, G = 1.5311, G_crit_ = 1.8871; no outliers detected).

It is recommended, as a precautionary measure, to set the cut-off for permissible retrograde calculation of the minimum blood alcohol concentration at 0.06‰. This threshold was not selected as a mere analytical limit of detection or quantification; rather, it is intrinsically linked to the structure and applicability range of the present model. Specifically, 0.06‰ represents the lowest BAC range for which a sufficient number of post-absorptive data points were available and for which stable regression-based elimination behaviour and model-based time corrections could be consistently demonstrated. The simulation-based approach, including Monte Carlo modelling, was explicitly developed and validated within this concentration domain (0.06–0.15‰), where elimination kinetics remained sufficiently predictable and the resulting correction factors showed robust behaviour. At the same time, this threshold lies clearly above the analytical detection and quantification limits of the applied forensic blood alcohol analysis, while remaining sufficiently low to capture legally relevant borderline constellations. In this concentration range, analytical imprecision and further factors may exert a non-negligible influence on the measured value, thereby limiting the robustness of any retrospective calculation, such that the additional uncertainty introduced by back-calculation outweighs the evidential value of the result. In addition, at very low blood alcohol concentrations, ethanol metabolism increasingly approaches first-order kinetics as enzymatic saturation diminishes, further reducing the predictability of elimination behaviour. Reported endogenous ethanol concentrations in human blood typically range between approximately 0.01 and 0.05‰, depending on methodological approach and reference source [9, 10]. Taken together, these considerations justify a conservative lower limit of 0.06‰ for retrograde calculation of minimum BAC as a data-supported and model-consistent lower boundary – one that balances empirical robustness, biological plausibility, analytical limitations, and forensic conservatism, rather than reflecting a purely technical analytical limit, thereby providing an appropriate safeguard against overinterpretation of analytically and physiologically unstable low-concentration findings.

### Consideration of BAC values below 0.15‰ for back-calculation

The descriptive statistical parameters of BAC elimination below 0.15‰ are shown in Table 2.

**Table 2:** Descriptive statistics of linear alcohol elimination below 0.15‰

Mean BAC decline [‰/h]		*n*
Study		
Wheat beer	0.075	15
Wheat beer + Eezup!	0.080	11
Apple wine	0.105	18
Grappa	0.072	9
Vodka	0.084	22
Total		75
Arithmetic mean x̄	0.083‰/h	
Median	0.080‰/h	
Standard deviation (SD)	0.030‰/h	
Variance	< 0.001(‰/h)²	
Minimum	0.028‰/h	
Maximum	0.200‰/h	
95% confidence interval (CI)	[0.076; 0.091]‰/h	
Standard error (SE)	± 0.004‰/h	
Margin of error (95%) = z x SE ; z = 1,96	± 0.007‰/h	

Using a Student’s t test with 74 degrees of freedom, it was assessed whether the mean elimination rate below 0.15‰ differs significantly from the conventionally conservative value of 0.1‰/h. The test evaluates the deviation of the arithmetic mean (x̄ = 0.083‰/h) from the reference value (µ₀ = 0.1‰/h) in units of the standard error:

$$\begin{array}{c}t\left(df\left(Degrees\;of\;Freedom\right)=n-1\right)\\=t\left(74\right)=\left(\overline x-\mu_0\right)/SE\approx-4;p<0.00001\end{array}$$

Thus, the deviation is highly significant, amounting to almost five standard deviations, with the true mean lying 4.8 times the standard error away from the reference value of 0.1‰/h.

This indicates that, although the average elimination rate below 0.15‰ (0.083‰/h) remains close to that of other intervals in the re-analysis using back-calculation simulation scenarios, the Student’s t test demonstrates a statistically significant deviation from the conservative value of 0.1‰/h. This finding justifies the need for alternative calculation models for the interval ≤ 0.15‰.

When evaluating new approaches for forensic back-calculation rules, it must be considered that, below 0.15‰, both the mean ethanol elimination rate of 0.083‰/h and the upper bound of the 95% confidence interval lie below the elimination rate of 0.1‰/h otherwise used as a minimum. In the conservative model, this value is applied; however, when starting from low BAC values, this may lead to a slight overestimation of elimination and, in individual cases, to incorrect retrograde BAC values. Since elimination rates exceed 0.1‰/h in all other segments of the curve, any disadvantage is considered minor.

To avoid this error, additional back-calculation rules were evaluated, either by using lower elimination rates or by compensating for the temporarily reduced elimination via shortening of the back-calculation time interval (time deduction).

In the realistic model, the mean elimination rate (r) of 0.083‰/h is applied, and a time adjustment to be subtracted (Δt_real_) is calculated according to the following equation:

General formula for back-calculation deduction:

$$\triangle t_{real}=\left(\left(0.1-r\right)/0.1\right)\times60$$

The upper and lower bounds of the 95% confidence interval were used as limits for the realistic range of elimination rates during back-calculation:

$$CI_{95\%}=\overline x+z\left(SD/\surd\right)$$

$$CI_{95\%\;real}=0.8342\pm1.96\left(0.02992/\surd75\right)=0.08342\pm0.00677$$

$$CI_{95\%\;real}=\left[0.7665;0.09019\right]$$

For the realistic model, the standard deviation of the mean elimination rate was ± 0.02992‰/h; the 95% confidence interval was [0.07665; 0.09019]‰/h, and the margin of error was ± 0.00677‰/h (margin of error = (CI_95% upper limit_ − CI_95% lower limit_)/2 = (0.09019‰/h − 0.07665‰/h)/2 = 0.00677‰/h). On this basis, the following realistic time deductions (*Δt*_*real*_) can be derived for retrograde calculation given an initial measured BAC within the respective concentration interval (see the first column of Table 3):

**Table 3:** Realistic time deductions (Δt_real_) for retrograde calculation of minimum BAC, stratified by measured BAC range

BAC range [‰]	Elimination rate *r* [‰/h]	Calculation (Δt_real_ = ((0.1 − *r*)/0.1) × 60)	∆t_real_ = realistic time reduction [min]
0.06–0.09	0.07665	(0.1–0.07665)/0.1 × 60	13.99
0.10–0.12	0.08342	(0.1–0.08342)/0.1 × 60	9.95
0.13–0.15	0.09019	(0.1–0.09019)/0.1 × 60	5.89

**Fig. 7:**
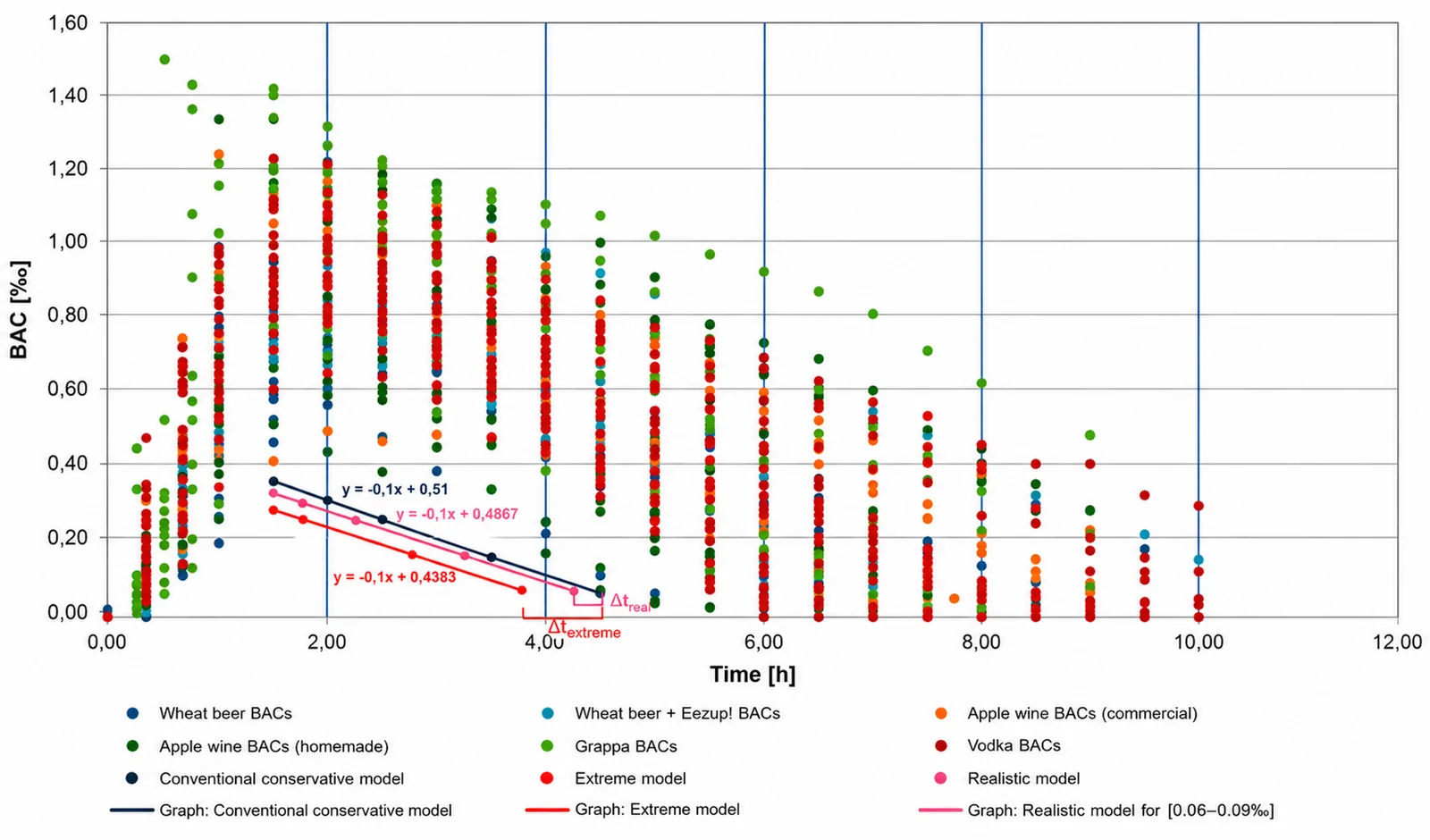
Single-point analysis of all measurement values including retrograde extrapolation simulation: conventional conservative model vs. realistic model vs. extreme model

Applying this model would entail subtracting approximately 14 min from the time interval between blood sampling and the incident for back-calculation of the minimum BAC when the measured BAC lies within [0.06–0.09‰]; a deduction of 10 min for BACs within [0.10–0.12‰]; and a deduction of 6 min when starting from [0.13–0.15‰]. This rule is intended to compensate for slower elimination at low BAC values and to prevent overestimation of BAC_retrograde_ without unduly restricting the applicability of retrograde calculation. It provides acceptable reliability, with a low margin of error and proportionate time deductions (see Fig. 7).

To validate this retrograde rule (“realistic model”), a Monte Carlo simulation with 1,000 iterations per BAC interval (as specified in the first column of the table) was performed. Inter-individual variability was incorporated by drawing, for each iteration, an elimination rate r from a normally distributed random distribution with mean x̄ = 0.08342‰/h and empirically determined standard deviation (SD) of 0.02992‰/h (*n* = 75). Using the standardised formula (see *General formula for back-calculation deduction*), the corresponding realistic back-calculation time deduction Δt_real_ was computed as described above.

Subsequently, for each of the three categories of initial BAC values ([0.06–0.09‰], [0.10–0.12‰], [0.13–0.15‰]), the corresponding empirical elimination rate r was applied as described in the Methods. The resulting Δt_real_ values were statistically evaluated (Table 4).

**Table 4:** Descriptive statistics of realistic back-calculation time deductions (Δt_real_) derived from Monte Carlo simulations

BAC range [‰]	Elimina-tion rate *r* [‰/h]	Mean ∆t_real_ [min]	SD ∆t_real_ [min]	Minimum ∆t_real_ [min]	Maximum ∆t_real_ [min]	95% CI [min]
0.06–0.09	0.07665	13.98	2.04	7.66	20.68	[10.10; 17.84]
0.10–0.12	0.08342	9.89	2.06	3.43	16.83	[5.97; 14.01]
0.13–0.15	0.09019	5.82	2.10	-1.39	11.51	[1.53; 9.84]

The Monte Carlo simulation results showed dispersion consistent with the analytically calculated time deductions and confirmed the methodological robustness and validity of the derived time deductions Δt_real_ across all three interval ranges. Moreover, the narrow confidence intervals provide a well-founded means of accounting for individual variability in retrograde BAC estimation.

The negative minimum observed in the range [0.13–0.15‰] is purely statistical in nature and results from isolated random draws with *r* > 0.1‰/h. In forensic practice, Δt_real_ would instead be set to 0 in such cases to avoid adding an artificial increase in the back-calculation interval.

Overall, these findings confirm the applicability of the retrograde model for measured BAC values < 0.15‰.

The realistic model was not intended as the final forensic recommendation, but as an intermediate derivative model to assess whether a differentiated, data-driven correction of the back-calculation interval is methodologically plausible. It demonstrates that the reduced elimination rate below 0.15‰ can be translated into proportionate time deductions that vary according to the measured BAC range. However, because forensic back-calculation of a minimum BAC must avoid any relevant risk of overestimation in disfavour of the subject, the realistic model primarily serves as a plausibility and validation step. The final practical recommendation is therefore based on the extreme model, which deliberately prioritises maximum forensic safety over precision.

The *extreme model*, which excludes any disadvantage to the affected individual due to even a slight overestimation of the back-calculated BAC, is based on the lowest elimination rate identified (0.02859‰/h). This was derived from the terminal low-concentration segment of the curve with the lowest elimination rate. Inspection of the corresponding BAC profile showed regular elimination behaviour above 0.15‰, with rates within the expected range. The markedly lower value in the terminal segment is attributable to minor fluctuations and a flattened regression. It therefore reflects late-phase variability rather than a biologically implausible elimination pattern. Accordingly, the extreme back-calculation deduction Δt_extreme_ is given as follows (Table 5):

**Table 5:** Extreme back-calculation time deduction (Δt_extreme_) based on lowest empirically observed ethanol elimination rate

BAC range [‰]	Most conservative elimination rate *r* [‰/h]	Calculation (∆t_extreme_ = ((0.1 – *r*)/0.1) × 60)	Extreme deduction ∆t_extreme_ [min]
0.06–0.15	0.02859	(0.1–0.02859)/0.1 × 60	42.85

For instance, if blood sampling was performed 5 h after the incident and the measured BAC was 0.06‰, the uncorrected conservative model would yield a retrograde BAC of 0.56‰ (0.06‰ + 0.1‰/h × 5 h). Under the realistic model, the applicable time deduction for the range 0.06–0.09‰ would be approximately 14 min, resulting in an effective back-calculation interval of 4 h 46 min and a retrograde BAC of approximately 0.54‰. Under the extreme model, the uniform deduction of 43 min would reduce the effective interval to 4 h 17 min, yielding approximately 0.49‰. This example illustrates that the realistic model provides a data-driven estimate of the expected correction, whereas the extreme model applies the maximum empirically justified deduction in favour of the subject.

Applying this one-off deduction from the back-calculation interval (Δt_extreme_) guarantees, based on the present data set, that not a single back-calculation result overestimates a true BAC, thereby ensuring an interpretation in favour of the subject. The most conservative elimination rate (*r* = 0.02859‰/h) represents the slowest elimination behaviour actually observed in the 75 valid curves analysed.

**Fig. 8:**
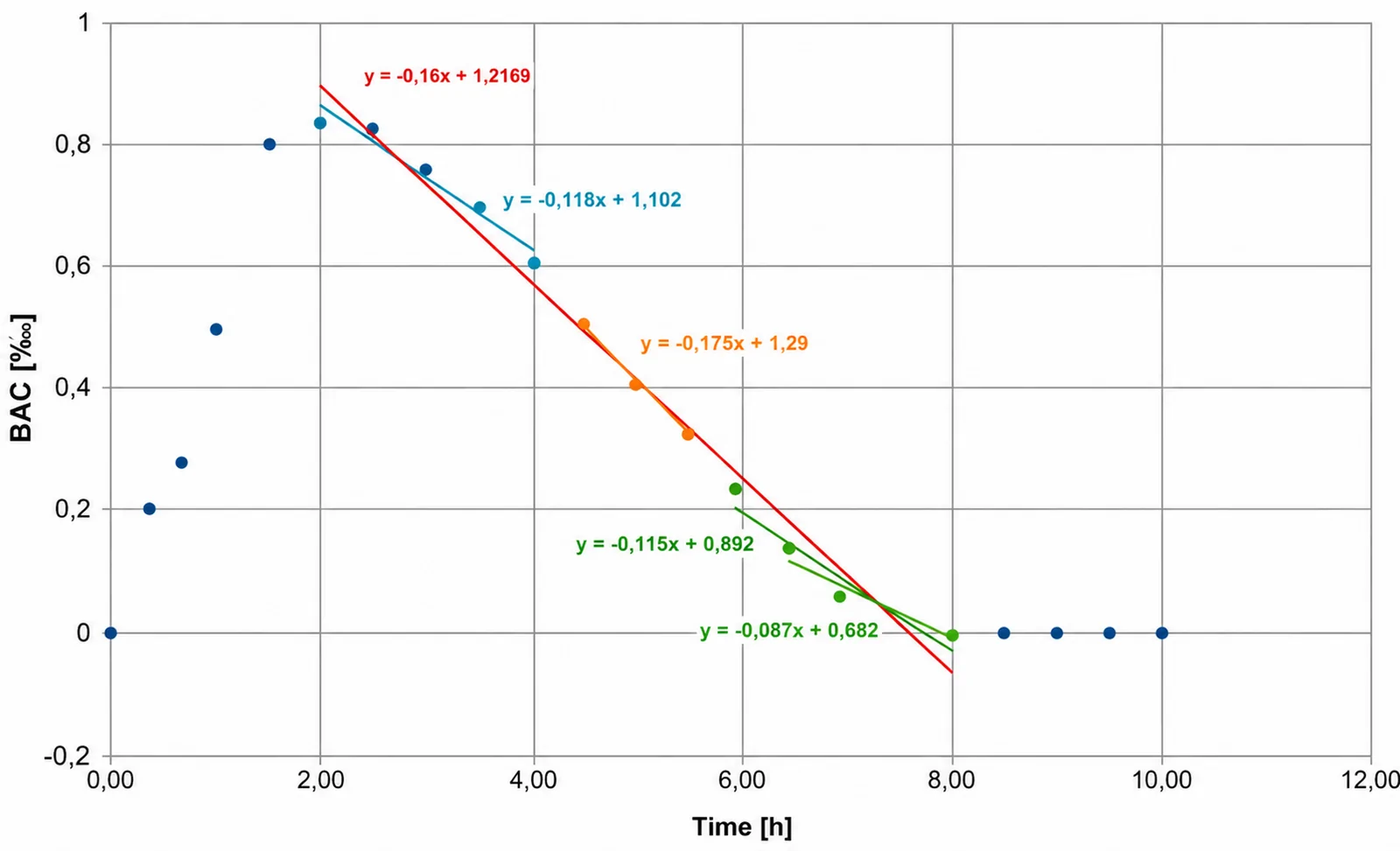
Example – Blood alcohol concentration-time curve with fitted regression lines

**Fig. 9:**
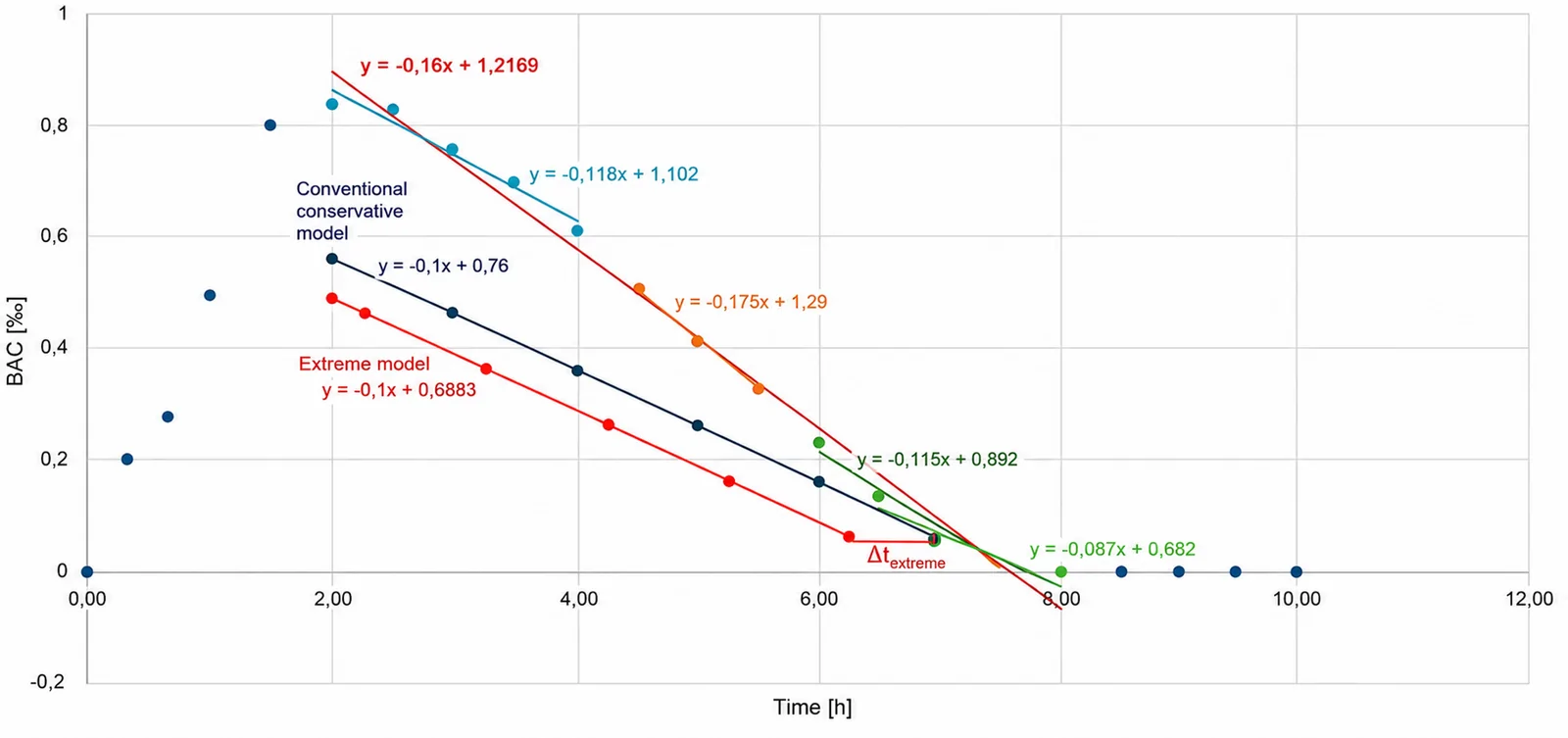
Example – Retrograde calculation using the conventional conservative model versus the extreme model, starting from 0.06‰ over 5 h

Consequently, a uniform time deduction of 43 min for retrograde calculation of the minimum BAC provides maximum forensic safety (see Figs. 7, 8 and 9).

As the extreme model is based on the lowest observed elimination rate and does not incorporate dispersion, a repeated Monte Carlo simulation to validate its results is not required. Although this model achieves maximum forensic safety, it will be overly restrictive in the vast majority of cases due to the large time deduction and may yield unrealistically low values of BAC_retrograde_. On the other hand, cases in which retrograde calculation would otherwise have been omitted below 0.15‰ are likely to involve scenarios requiring longer back-calculation intervals. Accordingly, the extreme model may open up additional possibilities for evaluation in such constellations.

In all models, back-calculation is performed using an hourly elimination rate of 0.1‰/h; however, in the realistic and extreme models, the corresponding subtractions from the relevant back-calculation intervals must additionally be applied.

The assumption of a linear relationship adopted for reasons of practicality appears justified on the basis of the present data sets. Accordingly, retrograde calculation of blood alcohol concentrations should also be scientifically comprehensible and defensible for measured values < 0.15‰, particularly when incorporating a time correction component.

## Conclusion

In some traditions and jurisdictions, for measured blood alcohol concentrations below 0.15‰, retrograde calculation to estimate offence-relevant BAC at the time of the incident is usually omitted, a practice that is historically rooted in uncertainties associated with earlier measurement techniques. Analysis of raw data from controlled drinking experiments with serial sampling to near-zero concentrations demonstrated comparable hourly elimination rates of approximately 0.13‰/h across different concentration intervals of the elimination curve.

In many forensic contexts, particularly when estimating minimum blood alcohol concentrations in favour of the subject, conservative elimination rates are applied, most commonly around 0.1‰/h. However, when examining alcohol elimination below 0.15‰ using linear regression, systematically lower elimination rates were observed. This transient underestimation can be compensated – while maintaining the minimum elimination rate – by shortening the back-calculation interval.

Thus, it is proposed that, for retrograde calculation of the minimum BAC at the time of the incident, a one-time deduction of 43 min be applied to the standard back-calculation interval for measured BAC values between 0.06 and 0.14‰, followed by continued calculation using an elimination rate of 0.1‰/h. Only for measured BAC values below 0.06‰ should retrograde calculation of the minimum BAC be omitted.

## Supplementary Information

Below is the link to the supplementary material.

Supplementary Material 1 (DOCX 31.4 KB)

## Data Availability

The datasets analysed during the current study are available from the corresponding author on reasonable request.
