# Supplementary material for: On the Possibility of retrograde calculation of blood alcohol concentrations below 0.15‰
Source: Int J Legal Med. 2026 May 28;140(5):2833–44. doi: 10.1007/s00414-026-03838-6 (PMC13499746; doi:10.1007/s00414-026-03838-6)
Supplement: Supplementary file 1 [file 414_2026_3838_MOESM1_ESM.docx]

*Supplementary Table S1:* Mean ethanol elimination rates below 0.15‰ by beverage type

*Supplementary Table S2:* Mean slopes of linear regression analyses for different blood alcohol concentration intervals and beverage types

**Supplementary Material**

*Supplementary Table S1*

| **Specific analysis for 0.15‰–0‰** |  |  |
| --- | --- | --- |
| **Type of beverage** | **Mean slope** | **n** |
| Wheat beer | -0.075466667 | 15 |
| Wheat beer + Eezup! | -0.079909091 | 11 |
| Apple wine (commercial and homemade) | -0.105453529 | 18 |
| Grappa | -0.071967778 | 9 |
| Vodka | -0.084310909 | 22 |
| **Overall mean / Total n** | **-0.083421595** | **75** |

*Supplementary Table S2*

| **Regression interval** | **Mean slope (wheat beer)** | **Mean slope (wheat beer  + Eezup!)** | **Mean slope (apple wine, commercial and homemade)** | **Mean slope (grappa)** | **Mean slope (vodka)** | **Row-wise mean of slopes** |
| --- | --- | --- | --- | --- | --- | --- |
| **c_max_ - 0‰** | -0.1427 | -0.1497 | -0.1622 | -0.1676 | -0.1592 | -0.15628 |
| **0.3 - 0,0‰** | -0.11065 | -0.12647 | -0.1419736 | -0.143 | -0.1245391 | **-0.129327563** |
| **0.6 - 0.3‰** | -0.161875 | -0.175 | -0.1740045 | -0.182272 | -0.1842666 | -0.175483788 |
| **0.9 - 0.6‰** | -0.1104933 | -0.10866 | -0.16268 | -0.14275 | -0.1385 | -0.132618 |
| **c_max_ - 0.6‰** | -0.10518 | -0.10383 | -0.154825 | -0.13475 | -0.132725 | -0.126262667 |
| **c_max_ - 0.9‰** | -0.078 | -0.1015 | -0.12133076 | -0.153625 | -0.06932 | -0.104755154 |
| **Column-wise mean of slopes** | -0.113239 | -0.1230 | -0.1509628 | -0.151279 | -0.129870 | -0.1374545  **———————————** -0.13368943 |
